# Intracellular Iron Deficiency and Abnormal Metabolism, Not Ferroptosis, Contributes to Homocysteine-Induced Vascular Endothelial Cell Death

**DOI:** 10.3390/biomedicines12102301

**Published:** 2024-10-10

**Authors:** Wenting Shi, Jing Zhang, Wairong Zhao, Meiyan Yue, Jie Ma, Silu Zeng, Jingyi Tang, Yu Wang, Zhongyan Zhou

**Affiliations:** 1 Longhua Hospital, Shanghai University of Traditional Chinese Medicine, Shanghai 200032, China; lh06058@shutcm.edu.cn (W.S.); witoy17@163.com (W.Z.); yuemiyan@126.com (M.Y.); ma_chieh@126.com (J.M.); julia24991@hotmail.com (S.Z.); dr_tang@163.com (J.T.); 2 State Key Laboratory of Pharmaceutical Biotechnology, The University of Hong Kong, Hong Kong SAR, China; jzhanghk@hku.hk; 3 Department of Pharmacology and Pharmacy, LKS Faculty of Medicine, The University of Hong Kong, Hong Kong SAR, China; 4 School of Acupuncture-Moxibustion and Tuina, Shanghai University of Traditional Chinese Medicine, Shanghai 201203, China

**Keywords:** homocysteinemia, endothelial dysfunction, apoptosis, autophagy, antioxidant system, iron deficiency

## Abstract

**Background/Objectives:** Homocysteine (Hcy) and iron are factors co-related with the progression of cardiovascular diseases. The vascular endothelium is an important barrier for physiological homeostasis, and its impairment initiates cardiovascular injury. However, the mechanism underlying Hcy-caused vascular endothelial cell injury and the participation of iron are not fully elucidated. This study aims to investigate the Hcy-induced vascular endothelial injury and iron metabolism dysfunction as well as the underlying molecular mechanism. **Methods:** Human umbilical vein endothelial cells (HUVECs) were employed as the experimental model to examine the Hcy-induced endothelial injury and its underlying mechanism via various biochemical assays. **Results:** Hcy suppressed the cell viability and proliferation and caused cell death in a concentration-dependent manner. Hcy induced cell cycle arrest, apoptosis, and autophagy as well as impairment of intracellular energy metabolism. Hcy disrupted the intracellular antioxidant system and mitochondrial function by increasing intracellular ROS, MDA and mitochondrial content, and decreasing the SOD activity and mitochondrial membrane potential. Hcy significantly reduced the GSH-Px activity along with the accumulation of intracellular GSH in a concentration-dependent manner. Ferroptosis inhibitors, Ferrostatin-1 (Fer-1), and Deferoxamine (DFO) significantly decreased the Hcy-caused cytotoxicity accompanied by a reduction in dysregulated mitochondria content, but only DFO ameliorated the elevation of intracellular ROS, and neither Fer-1 nor DFO affected the Hcy-caused reduction in intracellular ATP. In addition, Hcy decreased the intracellular concentration of iron, and supplementing Hcy with various concentrations of Fe^3+^ increased the cell viability and decreased the LDH release in a concentration-dependent manner. Hcy dramatically decreased the mRNA expression level of transferrin receptor while increasing the mRNA expression levels of transferrin, ferritin light chain, ferritin heavy chain, ferroportin, and SLC7A11. Moreover, Hcy suppressed the protein expression of phospho-Akt, phospho-mTOR, Beclin-1, LC3A/B, Nrf2, HO-1, phospho-MEK1/2, phospho-ERK1/2, and Caspase-3 in concentration- and time-dependent manners. **Conclusions:** Hcy-induced vascular endothelial injury is likely to be associated with apoptosis and autophagy, but not ferroptosis. The key underlying mechanisms are involved in the disruption of the intracellular antioxidant system and iron metabolism via regulation of PI3K/Akt/mTOR, MAPKs, Nrf2/HO-1, and iron metabolism.

## 1. Introduction

Hyper homocysteinemia (HHcy) is diagnosed by the elevation of the plasma homocysteine (Hcy) concentration over 15 µM, in which the patient’s plasma concentration of Hcy over 100 µM is classified as severe HHcy [1]. HHcy is an independent risk factor of common cardiovascular and central nervous disorders [2,3], in particular accelerating atherogenesis [4]. Almost 37.2% of people suffer from HHcy in China, and HHcy easily occurs in the Asian ethnicity and in males [5]. HHcy dramatically increases the incidence of cardiovascular disease by 79.1% [6], and the prevalence significantly increases along with aging [7]. Although supplementation with folic acid and vitamin B, which promote the metabolism of Hcy, decreases the plasma level of Hcy in clinics, several large-scale clinical studies have revealed that Hcy-lowering interventions do not reduce the incidence of cardiovascular events [8,9]. Thus, the physiological damage caused by Hcy and the understanding of its underlying mechanism of action are very important for HHcy therapy and need further investigation.

HHcy results in vascular endothelial dysfunction, which is considered as the essential pathological characteristic, and consequently accelerates the progression of cardiovascular disease [10]. The disruptions of vascular structure and microcirculation, which are closely related to vascular endothelial dysfunction, result from HHcy [10,11,12]. As we all know, vascular endothelial cells play a central role in the maintenance of vascular function. Hcy impairs the mitochondrial function and promotes intracellular reactive oxidative species (ROS) accumulation in vascular endothelial cells, thus contributing to cytotoxicity and cell death [10,13]. Hcy results in apoptosis [14,15] and autophagy [16], which are two types of classical programmed cell death, in vascular endothelial cells. However, previous studies have also suggested that HHcy might be beneficial for mitochondria homeostasis related to anti-oxidative defense [17] which indicates the anti-apoptotic effect of Hcy. Sato et al. found that amino acid starvation-induced autophagy trigged the Hcy-induced apoptosis in vascular endothelial cells [18]. The roles of both autophagy and apoptosis in Hcy-induced endothelial death are full of controversy, and the underlying molecular mechanisms have not been fully investigated.

Metal elements correlate with body health. Several previous studies have suggested an interaction between copper and Hcy-promoted cardiovascular disease [19,20]. Meanwhile, ferroptosis is a new form of programmed cell death which is characterized by intracellular lipid peroxidation along with the dysfunction of glutathione (GSH), glutamine-cysteine, and ferric metabolisms and transportations [21]. Iron catalyzed the formation of Hcy [22] and enhanced Hcy-induced vascular endothelial dysfunction [23], and the iron chelation was beneficial for HHcy [24]. Hcy induced up-regulation of ferritin types L and H in a Akt-dependent manner in human umbilical vein endothelial cells (HUVECs) [25]. Both iron overload and deficiency are correlated with the development of cardiovascular disease [22]. But whether endothelial iron metabolism and ferroptosis, a type of iron-dependent programmed cell death, contribute to Hcy-induced vascular endothelial cell death was not fully investigated.

In this study, we employed HUVECs as our in vitro model to evaluate iron metabolism and the different roles of programmed cell death, including apoptosis, autophagy, and ferroptosis, in Hcy-induced vascular endothelial toxicity. The action mechanisms underlying Hcy-induced vascular endothelial death were also partially elucidated.

## 2. Material and Methods

### 2.1. Chemicals and Reagents

Dulbecco’s modified Eagle’s medium (DMEM, Cat. No. 11965092), Fetal Bovine Serum (FBS, Cat. No. 10099141C), and penicillin/streptomycin (Cat. No. 10378016) were supplied by Thermo Fisher Scientific (Waltham, MA, USA). A cytotoxicity detection kit (LDH, Cat. No. 11644793001) was purchased from Roche (Mannheim, Germany). 3-(4,5-dimethyl-thiazol-2-yl)-2,5-diphenyl tetrazolium bromide (MTT, Cat. No. M2003), homocysteine (Hcy, Cat. No. H4628), and 3-Methyladenine (3-MA, Cat. No. M9281) were bought from Sigma Aldrich (St Louis, MO, USA). LY294002 (LY, Cat. No. S1737), wortmanin (Wort, Cat. No. S1952), rapamycin (Rapa, Cat. No. S1842), a Lipid Peroxidation MDA Assay Kit (Cat. No. S0131S), a Total Superoxide Dismutase Assay Kit with WST-8 (Cat. No. S0101S), a Total Glutathione Assay Kit (Cat. No. S0052), an ATP Assay Kit (Cat. No. S0026), a Cell Cycle and Apoptosis Kit (Cat. No. C1052), an Annexin V-FITC Apoptosis Detection Kit (Cat. No. C1062S), a Live/Dead Assay Kit (Cat. No. C2015S), a BeyoECL Moon chemifluorescence Kit (Cat. No. P0018FS), Mito-Tracker Green (Cat. No. C1048), dihydroethidium (DHE, Cat. No. S0063), and 5,5′,6,6′-Tetrachloro-1,1′,3,3′-tetraethyl-imidacarbocyanine (JC-1, Cat. No. C2005) were obtained from Beyotime Technology (Shanghai, China). Z-VDA(OH)-FMK (Cat. No. 14467), ferrostatin-1 (Fer-1, Cat. No. 17729), and deferoxamine (DFO, Cat. No. 14595) were obtained from Cayman chemical (Ann Arbor, Michigan, USA). TriPure Isolation Reagent (Cat. No. 11 667 165 001), a Transcriptor First Strand cDNA Synthesis Kit (Cat. No. 4897030001), and a FastStart Essential DNA Green Master Kit (Cat. No. 06924204001) were purchased from Roche Life Science (Mannheim, Germany). The primary antibodies Nrf2 (Cat. No. ab137550) and HO-1 (Cat. No. ab13248) were obtained from abcam (Cambridge, UK). The primary antibodies, including mTOR (Cat. No. 2983S), Phospho-mTOR (Cat. No. 5536s), Caspase-3 (Cat. No. 9665s), Beclin-1 (Cat. No. 3495T), LC3A/B (Cat. No. 12741T), Phospho-Akt (Cat. No. 2965s), Akt (Cat. No. 4685s), Phospho-ERK1/2 (Cat. No. 4370S), ERK1/2 (Cat. No. 4695s), Phospho-MEK1/2 (Cat. No. 9154S), MEK1/2 (Cat. No. 9122S), and GAPDH (Cat. No. 5174P), as well as the Secondary antibody horseradish peroxidase (HRP)-conjugated goat anti-rabbit IgG (Cat. No. 7074P2) were provided by Cell Signaling Technology (Danvers, MA, USA).

### 2.2. Cell Viability and Cytotoxicity Assay

Human umbilical vein endothelial cells (HUVECs, catalog: ATCC^®^ CRL-1730™) were purchased from ATCC, and the cell culture method was used, according to our previous study [26,27]. HUVECs were seeded in a 96-well plate at a density of 1 × 10^4^ cells/well and cultured for 24 h. Then, HUVECs were treated with various concentrations (1, 2, 4, 8 mM) of Hcy for 24 h, and the supernatant medium was transferred to another new 96-well plate followed by an LDH release assay using a cytotoxicity detection kit (LDH) according to the manufacturer’s instructions (Roche, Mannheim, Germany). The absorbance value of each 96-well plate at the wavelength of 490 nm was measured by a multiple-plate reader (M200, TECAN, Männedorf, Switzerland). The value of the cytotoxicity results was normalized to the folds of the control group. In addition, the attached cells were cultured with 1 mg/mL MTT solution for another 4 h and used for the cell viability assay. Then, the supernatant medium was discarded, and 100 µL DMSO was added into each 96-well plate. The absorbance value of each 96-well plate at the wavelength of 490 nm was measured by a multiple-plate reader (M200, TECAN, Männedorf, Switzerland). The value of cell viability was normalized to the percentage of the control group.

### 2.3. Real-Time Cell Proliferation and Cytotoxicity Analysis by xCELLigence

The attached HUVECs were recorded using a real-time cell analysis system (RTCA) named xCELLigence (S16, Agilent, Santa Clara, CA, USA) in a specific 16-well plate as described in our previous study [26,28]. For the evaluation of the anti-proliferation effect of Hcy, HUVECs were suspended in the cell culture medium containing various concentrations (2, 4, 8 mM) of Hcy for 24 h, and the number of attached cells was presented as the Cell Index. For evaluation of the real-time cytotoxicity effect of Hcy, HUVECs were cultured for 24 h and then treated with various concentrations (2, 4, 8 mM) of Hcy for another 24 h. The Cell Index was normalized to the folds of the value at the time point of adding Hcy.

### 2.4. Live and Dead Cell Staining

The live and dead cells were identified by calcein-AM and propidium iodide (PI) staining, respectively. HUVECs were seeded in the 96-well plate with a density of 1 × 10^4^ cells/well and further cultured to 70–80% confluence. Then, HUVECs were treated with Hcy (2, 4 and 8 mM) for 24 h. The HUVECs were stained by calcein-AM (green) and PI (red) according to the Calcein/PI Live/Dead Viability/Cytotoxicity Assay Kit manufacturer’s instructions (Beyotime, Shanghai, China). This was followed by a photograph using an inverted fluorescence microscope equipped with a 4 × objective. The ratio of live (green) and dead (red) cells was counted in each group.

### 2.5. Cell Cycle and Cell Apoptosis Analysis by Flow Cytometry

The cell cycle and apoptosis were analyzed by flow cytometry (CytoFLEX, Beckman, Brea, CA, USA). HUVECs were seeded in a 60 mm petri dish at a density of 50 × 10^4^ cell/dish and cultured for 24 h. Then, HUVECs were treated with or without Hcy (8 mM) for 24 h. Afterward, the cells were fixed with 70% ethanol at 4 °C overnight. The intracellular DNA was stained by propidium iodine (PI), followed by cell cycle analysis. The cell population percentages of G0/G1, S, and G2/M phases were summarized. For cell apoptosis analysis, after drug treatment, the HUVECs were double-stained with Annexin V-FITC and PI according to the Cell Cycle and Apoptosis Analysis Kit manufacturer’s instructions (Beyotime, Shanghai, China), and then the apoptotic cells were analyzed by flow cytometry (CytoFLEX, Beckman, Brea, CA, USA).

### 2.6. Intracellular MDA, SOD, GSH, ATP and Iron Assay

HUVECs were seeded in a 60 mm petri dish at a density of 50 × 10^4^ cell/dish and cultured for 24 h. Then, HUVECs were treated with various concentrations (2, 4, and 8 mM) of Hcy for 24 h. The intracellular malondialdehyde (MDA), superoxide dismutase (SOD) activity, glutathione (GSH), ATP and Iron were detected by the Lipid Peroxidation MDA Assay Kit (Beyotime, Shanghai, China), the Total Superoxide Dismutase Assay Kit with WST-8 (Beyotime, Shanghai, China), the Total Glutathione Assay Kit (Beyotime, Shanghai, China), the Intracellular ATP Detection Kit (Beyotime, Shanghai, China) and the tissue iron assay kit (Nanjing Jiancheng Bioengineering Institute, Nanjing, China) according to the manufacturer’s instructions, respectively. The results were normalized to the folds of the control group.

### 2.7. Detection of Intracellular ROS, Mitochondrial Content, and Membrane Potential

HUVECs were seeded in a 96-well plate at a density of 1 × 10^4^ cells/well and cultured for 24 h. Then, HUVECs were treated with Hcy (8 mM), with or without Fer-1 (80 µM) or DFO (80 µM), for 24 h, and followed by dihydroethidium (DHE) (Beyotime, Shanghai, China), Mito-Tracker Green (Beyotime, Shanghai, China), or JC-1 (Beyotime, Shanghai, China) staining, according to the manufacturer. The cell morphology was recorded by fluorescence microscopy. The intracellular contents of ROS and mitochondria were quantified by red and green fluorescence intensity, respectively. The mitochondrial membrane potential was presented as the ratio of green to red fluorescence intensity. All the values were normalized to the folds of the control group.

### 2.8. Real-Time PCR Analysis

HUVECs were seeded in a 60 mm petri dish at a density of 50 × 10^4^ cell/dish and cultured for 24 h. Then, HUVECs were treated with Hcy (8 mM) for 24 h. The interested genes were detected by real-time PCR analysis as per our previous description [29,30,31]. Briefly, the total RNA of each sample was collected using TriPure Isolation Reagent (Roche, Mannheim, Germany), and its RNA concentration was measured by a Microplate Reader (Infinite M200Pro NanoQuant, TECAN, Männedorf, Switzerland). The total RNA was transcribed to cDNA using the Transcriptor First Strand cDNA Synthesis Kit (Roche, Mannheim, Germany). The FastStart Essential DNA Green Master Kit (Roche, Mannheim, Germany) was used for real-time detection of the PCR product on the Light Cycle 96 platform (LC96, Roche, Mannheim, Germany), along with specific primers (Table 1). The PCR procedure was: 95 °C for 30 s, followed by 45 cycles of 95 °C for 5 s, 60 °C for 10 s, and 60 °C for 3 min. This was followed by melting curve analysis. The mRNA expression levels of the transferrin, transferrin receptor, ferritin light chain, ferritin heavy chain, ferroportin, and SLC7A11 genes were normalized to the internal control gene β-actin. The results were calculated using the 2^−∆∆Ct^ relative quantification method.

### 2.9. Western Blotting Analysis

After drug treatment, the protein expression levels of the interested targets were tested by Western blotting analysis as per our previous description [27,32]. HUVECs were washed with ice-cold PBS three times and lysed in a RIPA buffer, which contained 0.5 M NaCl, 50 mM Tris, 1 mM EDTA, 0.05% SDS, and 0.5% Triton X-100, supplemented with PMSF (1 mM) and phosphatase inhibitor (PhosStop, Roche, Mannheim, Germany), for 15 min on ice. Then, the cell lysates were centrifuged at 12,000× *g* for 15 min at 4 °C, followed by quantification of protein concentration using a BCA assay kit (Thermo Fisher Scientific, Waltham, MA, USA). Then, the protein was denatured at 95 °C for 5 min with 1× loading buffer, and 30 µg of total protein of each sample was separated by SDS-PAGE electrophoresis (Bio-Rad, Hercules, CA, USA). After protein separation, the proteins were transferred to a polyvinylidene fluoride (PVDF, 0.45 µm) membrane and then blocked with 5% skim milk for 2 h with gentle shaking at room temperature (RT). Immunoblots were incubated with primary antibodies, like Nrf2 (1:500), HO-1 (1:500), mTOR (1:1000), Phospho-mTOR (1:1000), Caspase-3 (1:1000), Beclin-1 (1:1000), LC3A/B (1:1000), Phospho-Akt (1:1000), Akt (1:1000), Phospho-ERK1/2 (1:1000), ERK1/2 (1:1000), Phospho-MEK1/2 (1:1000), MEK1/2 (1:1000), or GAPDH (1:2000), at 4 °C overnight. After washing with TBST three times, immunoblots were incubated with the secondary antibody horseradish peroxidase (HRP)-conjugated goat anti-rabbit IgG (1:2000) for 2 h at RT. Finally, immunoblots were visualized using an enhanced ECL system (BeyoECL moon, Beyotime, Shanghai, China). The immunoblots were then imaged on an imaging system (Amersham Imager 600, GE, Boston, MA, USA), and the integrated intensity of each protein band was analyzed by ImageJ software (1.49 V). The results were normalized with the internal control GAPDH and presented as the folds of the control group.

### 2.10. Statistical Analysis

Each experiment was replicated at least three times independently. Data were represented as the mean ± S.E.M. and analyzed by Graph Pad Prism 5.0 software. Statistical evaluation was performed using Student’s t-test or one-way ANOVA analysis. *p* < 0.05 was considered a significant difference.

## 3. Results

### 3.1. Homocysteine (Hcy)-Induced Vascular Endothelial Cell Death by Impairing Cell Viability and Cell Proliferation

**Figure 1:**
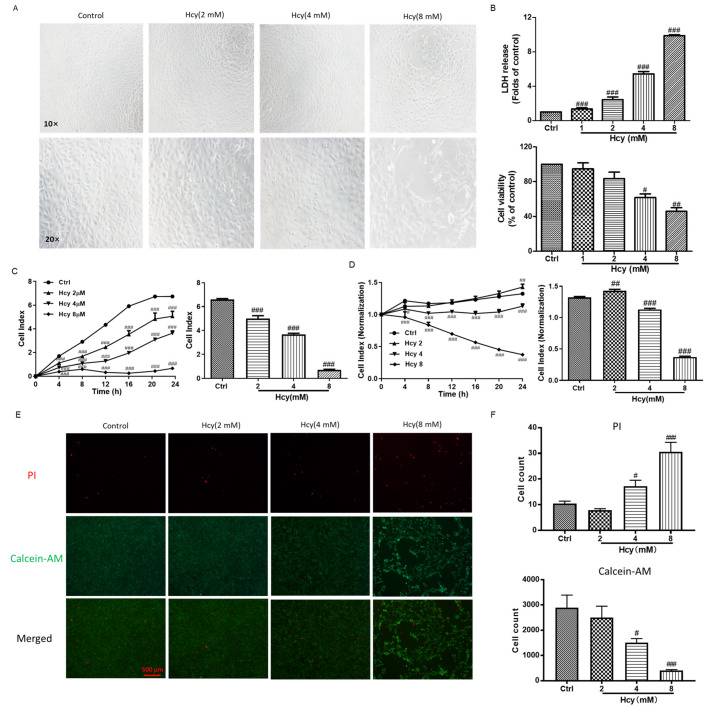
The effect of Hcy on endothelial cell morphology, cytotoxicity, and cell viability in HUVECs**.** (**A**) The cell morphology of HUVECs treated with indicated concentrations of Hcy for 24 h, n = 3. (**B**) The cytotoxicity and cell viability of Hcy were examined by LDH release and MTT assays, respectively. Data are presented as folds or percentages of the control group, n = 4. (**C**) HUVECs were suspended with various concentrations (2, 4, and 8 mM) of Hcy and cultured in an RTCA system for 24 h, and the anti-proliferation effect of Hcy is presented as the Cell Index. The Cell Index of the Hcy-treated group and the control group were also summarized at 24 h, n = 3. (**D**) HUVECs were cultured and attached for 24 h and then treated with various concentrations (2, 4, and 8 mM) of Hcy for another 24 h. The toxicity effect of Hcy was recorded in real time by RTCA. The Cell Index was normalized to the folds of the value at the time point of adding Hcy. The normalized Cell Index of the Hcy-treated group and control group were also summarized after treatment with Hcy for 24 h, n = 3. (**E**,**F**) Presentive images and analysis of live and dead cell staining. The green (calcein-AM) and red (PI) fluorescence demonstrate the live and dead cells, respectively, n = 5. Data are presented as mean ± S.E.M. # *p* ˂ 0.05, ## *p* ˂ 0.01, and ### *p* ˂ 0.001 vs. the control group.

Lactate dehydrogenase (LDH) release is a remarkable feature of cytotoxicity once the cell membranes are damaged [33]. Hcy affected the cell morphology and impaired the monolayer of vascular endothelial cells in HUVECs (Figure 1A). Hcy concentration-dependently induced LDH release, and the LDH level of the Hcy (8 mM)-treated group increased to ~10 folds of the control group (Figure 1B up). Hcy also decreased the cell viability in a concentration-dependent manner, and the cell viability of the Hcy (8 mM)-treated group decreased to ~45% of the control group (Figure 1B down). With the consistent results from the real-time cell analysis (RTCA) system, the HUVECs proliferation was significantly inhibited when suspended with various concentrations of Hcy (Figure 1C). Hcy caused cytotoxicity in the attached monolayer HUVECs, although Hcy slightly increased the Cell Index at the concentration of 2 mM (Figure 1D). The increased cell number or cell adhesion contributes to the elevation of the Cell Index in the RTCA system. Hcy (2 mM) might have increased the expression of adhesion molecules on the endothelial cell surface [34]. Moreover, according to the results from live and dead cell staining, Hcy concentration-dependently increased the number of dead cells and decreased the number of live cells in HUVECs (Figure 1E,F). These results indicate that Hcy inhibited cell proliferation and caused cell death in the vascular endothelial cells.

### 3.2. Hcy-Induced Vascular Endothelial Cell Death Involved in Cell Cycle Arrest, Apoptosis, and Autophagy as Well as Impairment of Energy Metabolism

**Figure 2:**
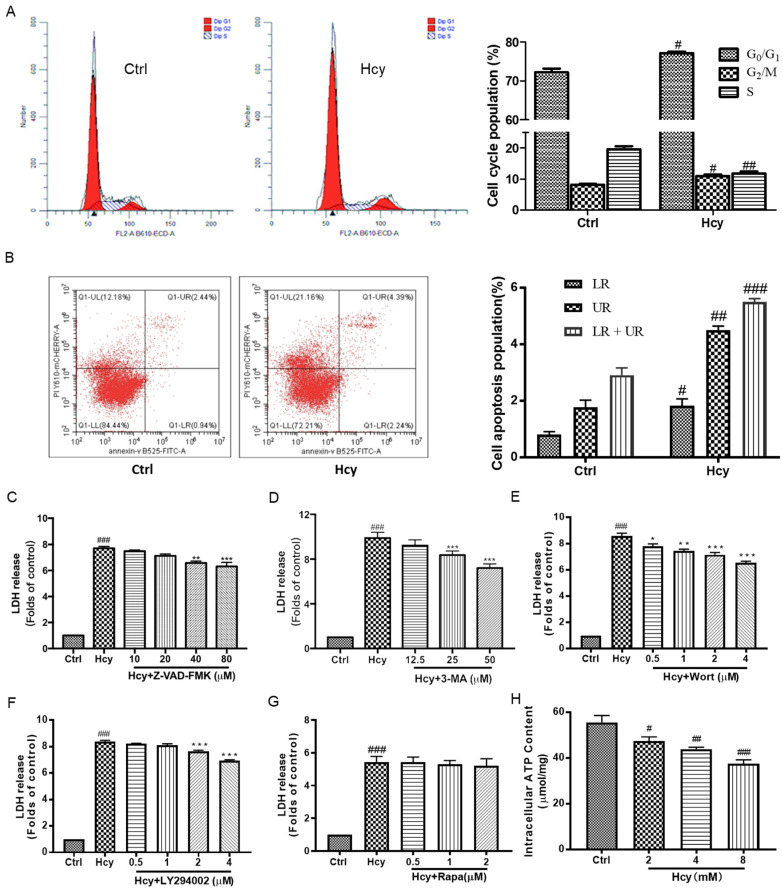
The effects of Hcy on cell cycle, apoptosis, autophagy, and energy metabolism in HUVECs. (**A**) The cell cycle was analyzed by PI staining, followed by flow cytometry. The cell population percentages of the G0/G1, S, and G2/M phases were summarized in both the control group and the Hcy-treated group, n = 3. (**B**) The apoptosis cells were detected by annexin V-FITC and PI double-staining using flow cytometry. The percentages of early (LR) and late (UR) apoptotic cells were calculated and summarized, n = 3. (**C**–**G**) HUVECs were co-treated with Hcy (8 mM) with various indicated concentrations of Z-VDA-FMK (n = 3), 3-MA (n = 3), Wort (n = 3), LY294002 (n = 3), or Rapa (n = 4) for 24 h. The cytotoxicity was examined by LDH release assay. Results are presented as folds of the control group. (**H**) The intracellular ATP concentration (µmol/mg protein) was quantified by the commercially available kit, n = 4. Data are presented as mean ± S.E.M. # *p* ˂ 0.05, ## *p* ˂ 0.01, and ### *p* ˂ 0.001 vs. the control group. * *p* ˂ 0.05, ** *p* ˂ 0.01, and *** *p* ˂ 0.001 vs. the Hcy-treated group.

Apoptosis and autophagy are well-studied programmed cell death forms [35]. The cell cycle analysis results, which were detected by flow cytometry, revealed that Hcy (8 mM) increased the cell number in the G0/G1 and G2/M phases, while it decreased the cell number in the S phase (Figure 2A). According to the results from cell apoptosis analysis via flow cytometry, Hcy significantly increased the population of apoptosis cells, including both early (LR) and later (UR) apoptosis stages (Figure 2B). Consistently, the co-treatment of various concentrations of apoptosis inhibitor Z-VAD-FMK with Hcy partially blocked Hcy-caused cytotoxicity in HUVECs (Figure 2C). Moreover, co-treatment of autophagy inhibitors, including 3-methyladenine (3-MA), wortmanin (Wort), and LY294002, with Hcy significantly attenuated Hcy-induced cell death (Figure 2D–F). Co-treatment of Hcy with the autophagy inducer rapamycin (Rapa), which is a potent and specific mTOR inhibitor, did not affect Hcy-caused cytotoxicity in HUVECs (Figure 2G). In addition, Hcy decreased the content of intracellular ATP in a concentration-dependent manner (Figure 2H). We concluded that Hcy-induced endothelial cell death was involved in cell cycle arrest, apoptosis, and autophagy, as well as intracellular energy metabolism. Autophagy might play an induction role in Hcy-caused endothelial cell death.

### 3.3. Hcy Disrupted the Intracellular Antioxidant System in Vascular Endothelial Cells

**Figure 3:**
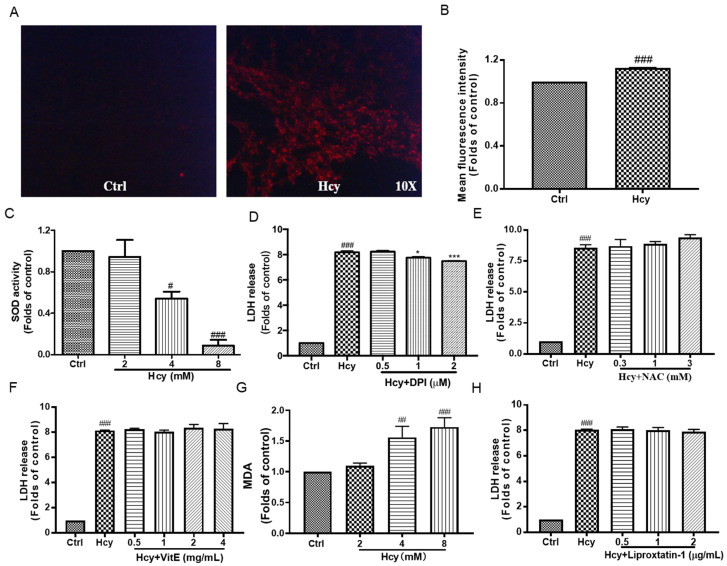
The effect of Hcy on intracellular ROS and lipid peroxidation in HUVECs. (**A**,**B**) The HUVECs were treated with Hcy (8 mM) for 24 h, and then the intracellular ROS were indicated by DHE staining. The fluorescence intensity was calculated using ImageJ software (1.49 V), n = 3. (**C**) The HUVECs were treated with various indicated concentrations of Hcy for 24 h and the SOD activity was measured using a commercially available kit, n = 3. (**D**–**F**) Co-treatment of Hcy (8 mM) with indicated concentrations of DPI (n = 3), NAC (n = 5), VitE (n = 3), and Liproxtatin-1 (n = 3) for 24 h, followed by cytotoxicity detection using LDH release kit. (**G**) The HUVECs were treated with various indicated concentrations of Hcy for 24 h. The intracellular level of MDA was measured using a commercially available kit, n = 8. (**H**) Co-treatment of Hcy (8 mM) with various indicated concentrations of Liproxtatin-1 for 24 h followed by LDH release assay, n = 3. The results were normalized to folds of the control group. Data are presented as mean ± S.E.M. # *p* ˂ 0.05, ## *p* ˂ 0.01, and ### *p* ˂ 0.001 vs. the control group. * *p* ˂ 0.05, and *** *p* ˂ 0.001 vs. the Hcy-treated group.

According to the results from dihydroethidium (DHE) staining (Figure 3A,B), Hcy significantly increased the fluorescence intensity in HUVECs, which indicated the elevation of intracellular reactive oxygen species (ROS). Hcy also decreased the SOD activity concentration-dependently (Figure 3C), and the co-treatment of Hcy with NADPH-oxidase inhibitor diphenyleneiodonium chloride (DPI) partially suppressed the cytotoxicity (Figure 3D). However, the co-treatment of Hcy with ROS scavengers, including N-acetylcysteine (NAC) and Vitamin E (VitE), did not affect the Hcy-induced cytotoxicity (Figure 3E,F). Hcy increased the level of malondialdehyde (MDA) to ~two-fold in HUVECs (Figure 3G). But the co-treatment of Hcy with various concentrations of Liproxtatin-1, which is a lipid peroxidation inhibitor and inhibits ferroptosis, did not ameliorate Hcy-induced cytotoxicity (Figure 3H). These results reveal that intracellular ROS contributed to Hcy-induced vascular endothelial cell toxicity partially in an NADPH-oxidase-dependent way. Hcy disrupted the intracellular antioxidant system and caused endothelial cell death, which cannot be reversed by targeted scavenging of ROS or lipid peroxidation.

### 3.4. Ferroptosis Might Not Participate in Hcy-Induced Vascular Endothelial Death

**Figure 4:**
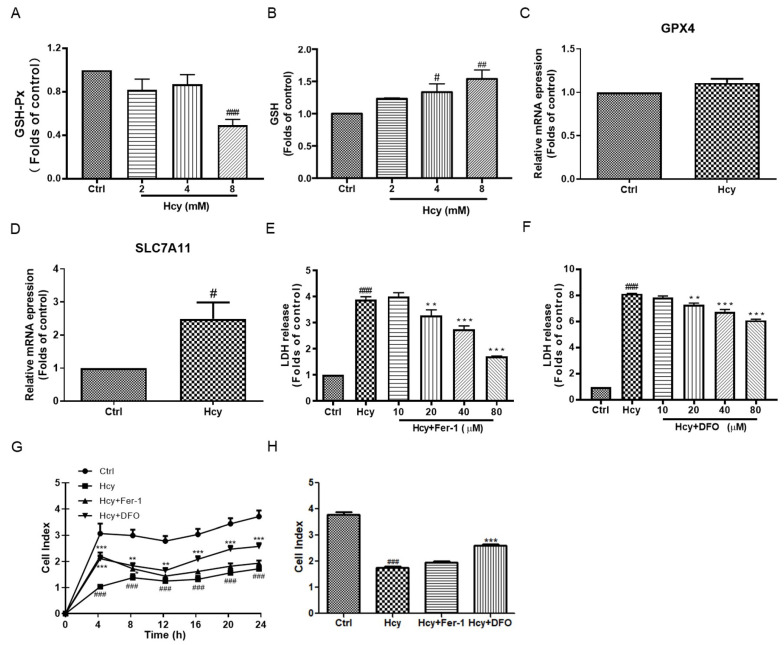
The role of ferroptosis on Hcy-induced vascular endothelial cell toxicity in HUVECs. (**A**,**B**) The HUVECs were treated with various concentrations (2, 4, and 8 mM) of Hcy for 24 h. The intracellular GSH-Px activity and GSH were measured by commercially available kits, n = 5. (**C**,**D**) The HUVECs were treated with Hcy (8 mM) for 24 h. The mRNA expression of GPX4 (n = 7) and SLC7A11 (n = 5) genes was examined by real-time PCR. (**E**,**F**) The HUVECs were co-treated with Hcy (8 mM), with various indicated concentrations of Fer-1 or DFO for 24 h, followed by the LDH release assay, n = 3. (**G**,**H**) HUVECs were suspended with Hcy (8 mM), with or without Fer-1 (80 µM) or DFO (80 µM), for 24 h. The cell proliferation was recorded in real time by RTCA, n = 4. The results were normalized to folds of the control group. Data are presented as mean ± S.E.M. # *p* ˂ 0.05, ## *p* ˂ 0.01, and ### *p* ˂ 0.001 vs. the control group. * *p* ˂ 0.05, ** *p* ˂ 0.01, and *** *p* ˂ 0.001 vs. the Hcy-treated group.

Iron metabolism is important for cardiovascular health, while its participation in Hcy-initiated cardiovascular damage is not fully elucidated [22]. Ferroptosis is a new form of programmed cell death that is characterized by lipid peroxidation and decreased glutathione peroxidase (GSH-Px) activity [21]. Hcy (8 mM) significantly decreased the GSH-Px activity (Figure 4A), along with the accumulation of intracellular GSH, in a concentration-dependent manner (Figure 4B), not affecting the mRNA expression of GSH-Px (GPX4) (Figure 4C). The mRNA expression of SLC7A11, which imports cysteine into cells and promotes GSH synthesis, was also increased by Hcy incubation in HUVECs (Figure 4D). Moreover, the co-treatment of Hcy with various concentrations of ferroptosis inhibitors, including Fer-1 (inhibition of lipid peroxidation) and DFO (iron chelator), significantly decreased the Hcy-caused cytotoxicity in HUVECs (Figure 4E,F). Only DFO partially protected the cells from Hcy-caused impairment of endothelial cell proliferation (Figure 4G,H) and migration (Figure S1).

**Figure 5:**
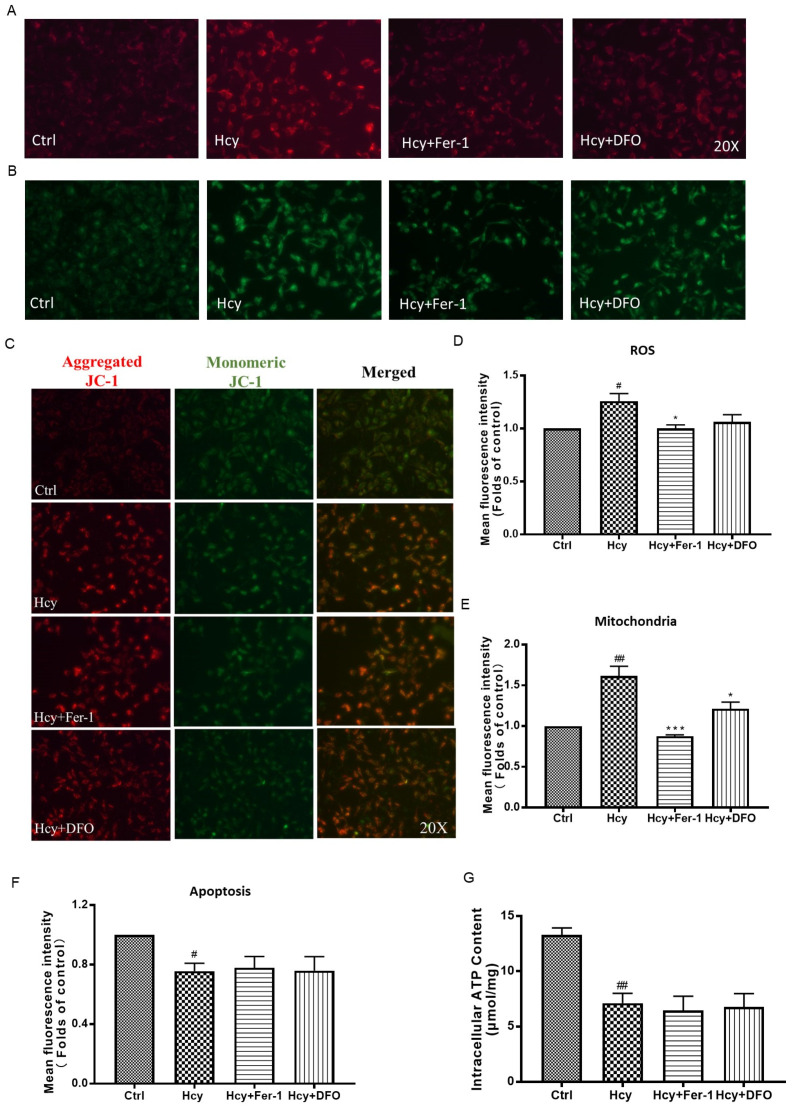
The effect of ferroptosis inhibitors on Hcy-induced intracellular ROS production and mitochondrial dysfunction in HUVECs. (**A**–**C**) The HUVECs were treated with Hcy (8 mM), with or without Fer-1 (80 µM) or DFO (80 µM), for 24 h. Then, the intracellular ROS, number of mitochondria, and mitochondria membrane potential were indicated by DHE (**A**), Mito-tracker (**B**), and JC-1 (**C**) staining, respectively, n = 3. (**D**–**F**) The fluorescence intensity was calculated using ImageJ software. The results were normalized to folds of the control group. (**G**) The HUVECs were treated with Hcy (8 mM), with or without Fer-1 (80 µM) or DFO (80 µM), for 24 h. Then, the intracellular concentration of ATP was detected by a commercially available kit, n = 4. Data are presented as mean ± S.E.M. # *p* ˂ 0.05 and ## *p* ˂ 0.01 vs. the control group. * *p* ˂ 0.05, and *** *p* ˂ 0.001 vs. the Hcy-treated group.

Moreover, Hcy increased the intracellular ROS production and mitochondrial content and decreased the mitochondrial membrane potential in HUVECs, while co-treatment of Hcy with ferroptosis inhibitors, including Fer-1 and DFO, significantly ameliorated the elevation of intracellular ROS and the mitochondrial number regardless of the mitochondrial membrane potential or the intracellular content of ATP (Figure 5). These results suggest that Hcy increased the number of mitochondria with impaired energy metabolism function in vascular endothelial cells. Ferroptosis might not be involved in Hcy-induced vascular endothelial death.

### 3.5. Hcy Caused Intracellular Iron Deficiency and Abnormal Iron Metabolism in Vascular Endothelial Cells

**Figure 6:**
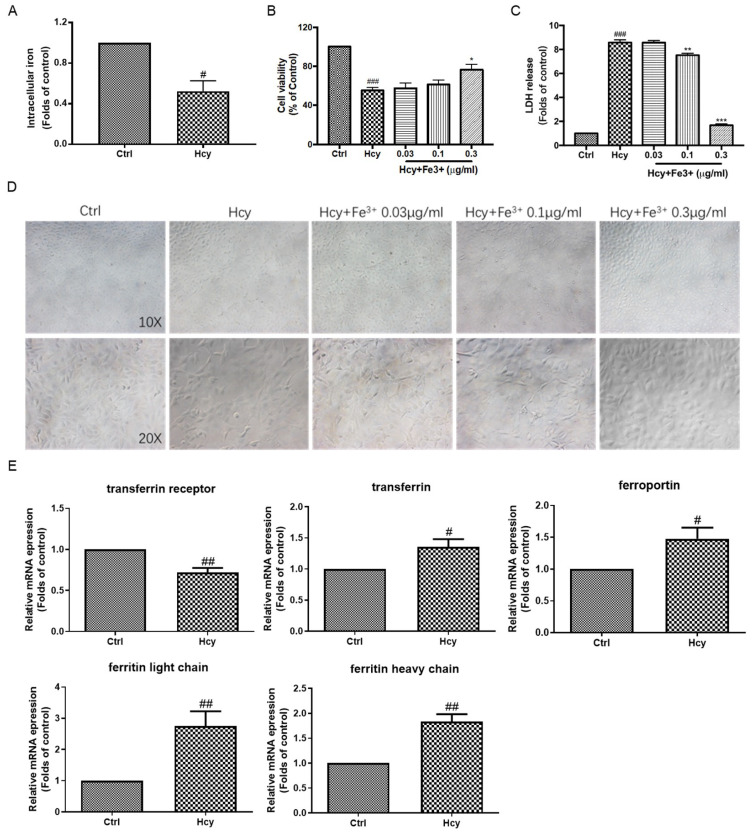
The role of iron metabolism in Hcy-induced vascular endothelial cell toxicity in HUVECs. (**A**) HUVECs were treated with Hcy (8 mM) for 24 h. The intracellular level of iron was measured by a commercially available kit according to its manual, n = 3. (**B**,**C**) Co-treatment of Hcy (8 mM) with indicated concentrations of Fe^3+^ for 24 h, followed by cell viability and cytotoxicity detections using MTT and LDH release assays, respectively, n = 3. (**D**) The cell morphology was observed by an inverted microscope equipped with 10× and 20× objective lenses. (**E**) The HUVECs were treated with or without Hcy (8 mM) for 24 h. The mRNA expressions of transferrin receptor (n = 6), transferrin (n = 5), ferritin light chain (n = 5), ferritin heavy chain (n = 6), and ferriportin (n = 6) genes were examined by real-time PCR. Results are presented as folds or percentages of the control group. Data are presented as mean ± S.E.M. # *p* ˂ 0.05, ## *p* ˂ 0.01, and ### *p* ˂ 0.001 vs. the control group. * *p* ˂ 0.05, ** *p* ˂ 0.01, and *** *p* ˂ 0.001 vs. the Hcy-treated group.

Ferroptosis is characterized by the accumulation of intracellular iron and dysfunction of the iron metabolism. In HUVECs, we found that treatment with Hcy (8 mM) decreased the intracellular concentration of iron to ~0.52-fold lower that of the control group (Figure 6A). Co-treatment of Hcy (8 mM) with various concentrations of Fe^3+^ (0.03–0.3 µg/mL) increased the cell viability and decreased the LDH release in a concentration-dependent manner (Figure 6B,C). Consistently, co-treatment of Hcy (8 mM) with Fe^3+^ (0.3 µg/mL) significantly maintained the monolayer of vascular endothelial cells (Figure 6D). In addition, Hcy dramatically decreased the mRNA expression level of transferrin receptor and increased the mRNA expression levels of transferrin, ferritin, and ferriportin (Figure 6E), which are key iron transportation- and metabolism-related genes. These results indicate that the intracellular iron deficiency and dysregulation of iron metabolism might be involved in Hcy-induced vascular endothelial toxicity regardless of ferroptosis.

### 3.6. Hcy Suppressed the Akt/mTOR, MAPKs, and Nrf2/HO-1 Signaling in Vascular Endothelial Cells

**Figure 7:**
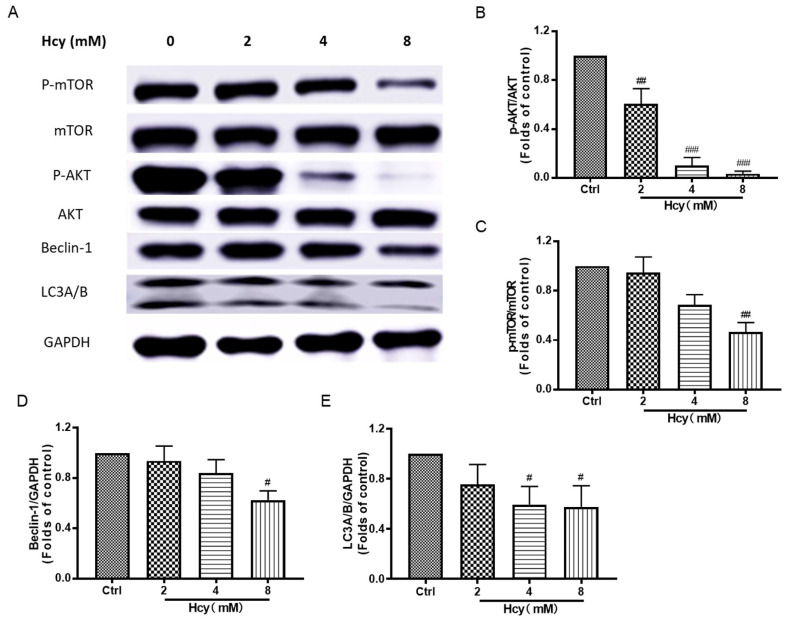
The effect of Hcy on Akt/mTOR autophagy signaling in HUVECs. (**A**) The HUVECs were treated with various concentrations (2, 4, and 8 mM) of Hcy for 24 h. The representative bands in Western blotting analysis. (**B**–**E**) The quantitative protein expressions of phospho-mTOR (n = 3), mTOR (n = 3), phospho-Akt (n = 4), Akt (n = 4), Beclin-1 (n = 4), LC3A/B (n = 4), and GAPDH (n = 4) were detected by Western blotting analysis. GAPDH served as the internal control. The results were normalized to folds of the control group. Data are presented as mean ± S.E.M. # *p* ˂ 0.05, ## *p* ˂ 0.01, and ### *p* ˂ 0.001 vs. the control group.

**Figure 8:**
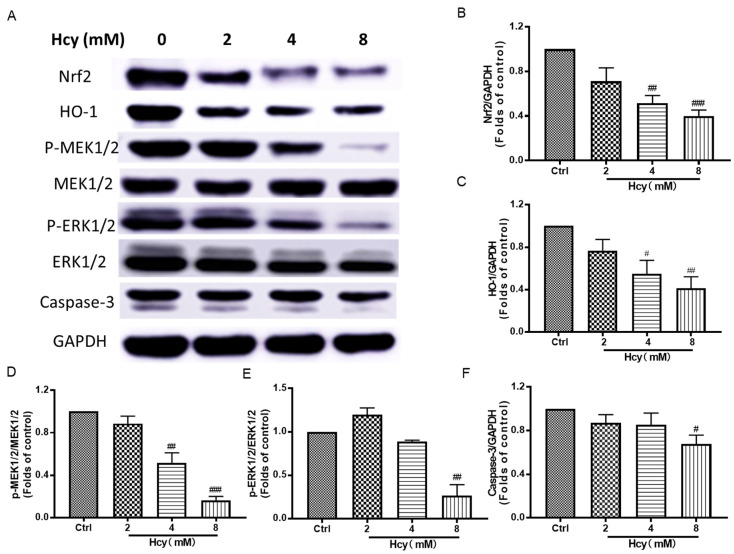
The effect of Hcy on MAPKs and Nrf2/HO-1 signaling in HUVECs. (**A**) The HUVECs were treated with various concentrations (2, 4, and 8 mM) of Hcy for 24 h. The representative bands in Western blotting analysis. (**B**–**F**) The quantitative protein expressions of Nrf2 (n = 4), HO-1 (n = 4), phospho-MEK1/2 (n = 4), MEK1/2 (n = 4), phospho-ERK1/2 (n = 3), ERK1/2 (n = 3), Caspase-3 (n = 4), and GAPDH (n = 3) were detected by Western blotting analysis. GAPDH served as the internal control. The results were normalized to folds of the control group. Data are presented as mean ± S.E.M. # *p* ˂ 0.05, ## *p* ˂ 0.01, and ### *p* ˂ 0.001 vs. the control group.

According to the results from Figure 2, autophagy was involved in Hcy-induced vascular endothelial damage. We further found that Hcy suppressed the protein expression of phospho-Akt, phospho-mTOR, Beclin-1, and LC3A/B in HUVECs (Figure 7), which are key proteins in Akt/mTOR signaling cascade and promote autophagy, in a concentration-dependent manner [16]. The ratio of phospho-Akt to total Akt was ~0.2-fold lower than that of the control group in the Hcy (8 mM)-treated group. In addition, MAPKs and Nrf2/HO-1 signaling are vital in the maintenance of the intracellular redox homeostasis system [36]. Treatment with Hcy dramatically decreased the protein expressions of Nrf2, HO-1, phospho-MEK1/2, phospho-ERK1/2, and Caspase-3 in a concentration-dependent manner in HUVECs (Figure 8), which are correlated with the cellular self-protective mechanism under oxidative stress. In accordance with these results, treatment with Hcy (8 mM) for 6, 12, or 24 h also suppressed Akt/mTOR, MAPKs, and Nrf2/HO-1 signaling in a time-dependent manner (Figures S2 and S3). However, the autophagy and apoptosis markers, including LC3A/B and Caspase-3, were also decreased in the Hcy-treated group, which might be related to the dramatic toxicity induced by supra-physiologically high concentration of Hcy. These results reveal that Hcy-caused cell death and intracellular oxidative stress might be involved in the suppression of Akt/mTOR, MAPKs, and Nrf2/HO-1 signaling.

## 4. Discussion

Hyper homocysteinemia (HHcy) has been connected with various diseases, especially in atherosclerosis; hypertension; and cognitive decline, primarily dementia [37]. In this study, we found that apoptosis and autophagy, but not ferroptosis, contributed to Hcy-caused endothelial cell death. The toxicity of Hcy resulted from elevated intracellular ROS, mitochondrial dysfunction, and abnormal iron metabolism.

Previous studies have shown that Hcy inhibits angiogenesis in both HUVECs and zebrafish embryos, and the underlying mechanism may occur through VEGF/VEGFR, Akt, and ERK1/2 inhibition [38], as well as cytoskeleton remodeling [39]. In endothelial cell HUVECs, we also found that Hcy impaired the cell morphology, inhibited the cell proliferation, and caused cell death in vascular endothelial cells (Figure 1). Hcy promoted apoptosis and arrested the progression of the cell cycle in vascular endothelial cells [40,41]. Consistently, Hcy caused cell cycle G_1_/S phase arrest (Figure 2A) and cell apoptosis (Figure 2B). These results indicate that Hcy-caused endothelial cell death is correlated with its impairment of cell viability, cell proliferation, and cell cycle progression, as well as the induction of cell apoptosis. Moreover, pharmacological inhibition of apoptosis (Figure 2C) and autophagy (Figure 2D–F) attenuated Hcy-induced endothelial cell death, while the induction of autophagy (Figure 2G) did not affect Hcy-caused cytotoxicity. But Zhang Y et al. proved that the autophagy inhibitor 3-methyladenine increases Hcy-induced endothelial cell senescence and that the autophagy inducer rapamycin alleviates Hcy-induced endothelial cell senescence [42]. Sato et al. found that both inhibition and induction of autophagy, by its inhibitor, chloroquine, and amino acid starvation, respectively, increased bovine aortic endothelial cell death [18]. Induction of autophagy enhanced Hcy-induced endothelial apoptosis, while inhibition of apoptosis blocked the autophagy-enhanced endothelial toxicity of Hcy [18]. Overall, autophagy is a double-edge sword in cell death according to our data, and it might be a cause of cell death in Hcy-induced endothelial cell toxicity. Hcy also decreased the intracellular ATP content in vascular endothelial cells (Figure 2H). Thus, Hcy-induced endothelial cell death was involved in the promotion of apoptosis and the disruption of intracellular energy metabolism. Autophagy plays an induction role in Hcy-induced endothelial cell death.

As we all know, ROS and lipid peroxide are associated with oxidative stress, which causes endothelial injury and, potentially, endothelial dysfunction [43]. In line with this conclusion, in our study, Hcy significantly elevated the intracellular ROS (Figure 3A,B) and decreased the SOD activity (Figure 3C). Previous studies have suggested that the suppression or scavenging of intracellular ROS might be promising strategies to block Hcy-caused endothelial damage [44]. We found that the pharmacological inhibition of NADPH-oxidase, which mediates the generation of ROS, partially suppressed the cytotoxicity of Hcy in vascular endothelial cells (Figure 3D). However, other ROS scavengers, including NAC and VitE, did not affect the Hcy-induced cytotoxicity (Figure 3E,F). Intracellular ROS results in lipid peroxidation [45]. Consistently, Hcy increased the intracellular level of MDA (Figure 3G), while the inhibition of lipid peroxidation did not ameliorate Hcy-induced endothelial cytotoxicity (Figure 3H). These results reveal that Hcy-induced vascular endothelial cell injury resulted from the elevation of intracellular ROS. Lipid peroxidation might be the consequence of ROS accumulation. And targeting the not-yet-identified cause of ROS accumulation might be promising for the amelioration of Hcy-caused vascular endothelial death.

MDA, GSH, and SOD activity are classical bio-markers of intracellular redox balance [46,47]. In this study, Hcy significantly decreased SOD activity (Figure 3C) and increased the intracellular MDA (Figure 3G) in vascular endothelial cells. But the intracellular GSH level was elevated after Hcy treatment along with decreased GPX4 activity, without a change in its mRNA expression (Figure 4A–C). Hcy induced increased mRNA expression of SLC7A11 (Figure 4D), which is a cysteine transporter and promotes the synthesis of GSH, and this might be the reason for the elevation of the intracellular GSH level. But the overexpression of SLC7A11 might have also contributed to cell death through toxic buildup of intracellular cystine and other disulfide molecules [48]. SLC7A11 exerted multiple pathological effects in inflammatory disease and cancers [49], although SLC7A11 resisted ferroptosis [50]. The SLC7A11 inhibitor significantly suppressed Hcy-induced vascular endothelial toxicity [18]. These results revealed that Hcy caused a disruption of intracellular redox homeostasis in endothelial cells. The elevation of SLC7A11 exerted a pathological effect in Hcy-induced endothelial toxicity, which might also indicate a suppression of ferroptosis by Hcy in vascular endothelial cells.

Apart from apoptosis and autophagy, ferroptosis is a new form of programmed cell death that is characterized by lipid peroxidation and iron overload [51]. The pharmacological inhibition of ferroptosis by the lipid peroxidation inhibitor Fer-1 and iron chelator DFO attenuated the Hcy-induced endothelial toxicity (Figure 4E,F). DFO promoted cell proliferation (Figure 4G,H) and cell migration (Figure S1). Many studies have shown a strong association between ferroptosis and mitochondrial dysfunction, disruption of ATP synthesis, and a subsequent increase in ROS levels, leading to abnormal iron transport and lipid peroxidation [52]. In this study, Fer-1 decreased intracellular ROS, and both Fer-1 and DFO decreased intracellular abnormal mitochondria content without amelioration of mitochondrial function, including mitochondrial membrane potential and ATP generation (Figure 5). These results were consistent with a previous study that found that iron chelation was beneficial for HHcy [24]. The inhibition of lipid peroxidation and iron might suppress Hcy-induced endothelial mitochondrial dysfunction and toxicity. In order to clarify whether the feature of ferroptosis exists in Hcy-treated endothelial cells, we measured the intracellular iron content and found that Hcy caused intracellular deficiency in endothelial cells and that the supplementation of iron improved the endothelial cell viability and monolayer morphology (Figure 6A–D). Iron homeostasis is important for normal physiological function and survival of cells [53]. These results suggest that intracellular iron deficiency is a pathological feature of Hcy-induced endothelial damage. Meanwhile, Hcy enhanced the mRNA expression of key protein-related iron metabolism (Figure 6E), which might be a self-protective mechanism of cells that accelerates the elevation of intracellular iron content against iron deficiency-triggered cell damage. Thus, Hcy did not induce endothelial cell ferroptosis through the promotion of iron overload, which was also proven by the elevation of the intracellular GSH and mRNA levels of ferroptosis inhibitor SLC7A11. In contrast, Hcy caused intracellular iron deficiency and abnormal iron metabolism, resulting in vascular endothelial death.

**Figure 9:**
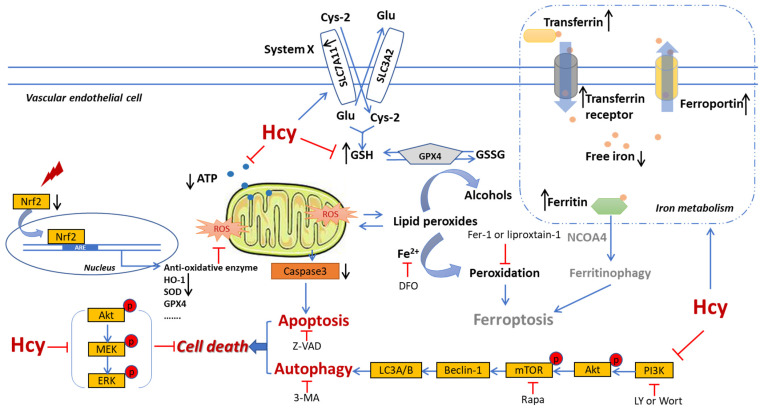
Schematic overview of the underlying mechanism of Hcy-induced endothelial death.

For the clarify of the molecular mechanism underlying Hcy-induced endothelial cell death, we mainly focused on the disruption of the intracellular anti-oxidative system, apoptosis, and autophagy according to the previous data and study [54]. PI3K/Akt/mTOR MAPKs and Nrf2/HO-1 signaling are involved in oxidative stress and cell survival in vascular endothelial cells [55,56]. In this study, Hcy inhibited cell survival and anti-oxidative signaling, and also decreased apoptosis and autophagy markers in HUVECs (Figure 7, Figure 8, Figures S2 and S3). These results indicate that Hcy might induce dramatic endothelial toxicity. PI3K/Akt/mTOR, MAPKs, and Nrf2/HO-1 signaling, as well as their regulated anti-oxidative system, apoptosis, and autophagy, were involved in the regulation of Hcy-induced vascular endothelial cell death (Figure 9).

This study provides evidence for the underlying mechanisms of HHcy-caused endothelial injury and emphasizes the iron deficiency and abnormal metabolism. HHcy is an independent risk factor for cardiovascular and cerebral vascular diseases. These findings will be beneficial for the understanding of the development of HHcy-associated diseases, for example, atherosclerosis, stroke, and H-type hypertension. Targeting iron metabolism might be promising for the discovery of drug candidates or interfering approaches to HHcy-associated diseases. The main challenge and limitation of this study is the concentration range of Hcy used for exploration of the effect of endothelial injury. The inhibition of endothelial proliferation has been reported at high Hcy concentrations (10 to 200 µM with a maximum of 1000 µM). While high concentrations of Hcy (~200 µM) produce endoplasmic reticulum (ER) stress in endothelial cell cultures, caspase-dependent cell death can be seen at 500–1000 µM. Various types of endothelial cell, such as EA.hy926, HUVECs, porcine aortic endothelial cell and mice aortic endothelial cell, etc., have been employed as in vitro models for the evaluation of Hcy-caused endothelial injury in previous studies. Similarly, the concentrations and treatment durations of Hcy were different in these studies. In addition, the passage number of cells, cell density, and detection method affect the Hcy-induced endothelial injury. In our study, we found concentration-dependent toxic concentrations of Hcy (1–8 mM) by simply measuring the cell viability and cytotoxicity using MTT and LDH release assays, and this might allow for new insights into the effects of supra-physiologically high concentrations of Hcy on endothelial injury. Whether other signaling pathways, such as eNOS, are affected by high concentrations of Hcy, as well as the effects of well-known Hcy-lowering agents vitamin B12 and folic acid on Hcy-induced endothelial injury, will be examined in the future.

## 5. Conclusions

In summary, apoptosis and autophagy, but not ferroptosis, mainly contribute to Hcy-induced endothelial cell death. Apart from the disruption of the intracellular antioxidant system and the accumulation of intracellular ROS, iron deficiency and iron abnormal metabolism play vital roles in Hcy-caused vascular endothelial cell injury.

## Figures and Tables

**Table 1:** The specific primers of related genes used in real-time PCR analysis.

Gene	Forward Primer	Reverse Primer
transferrin	5′-AGCCTGCACTTTCCGTAGAC-3′	5′-AACCACTTGGGCCAGTGAAA-3′
transferrin receptor	5′-GTTAGGGGCCGCCATCC-3′	5′-AAGAACACTAGCGCGTCCTC-3′
ferritin light chain	5′-CACCTGACCAACCTCCACAG-3′	5′-CGTGCTTGAGAGTGAGCCTT-3′
ferritin heavy chain	5′-ACTTTGACCGCGATGATGTG-3′	5′-CCTGAAGGAAGATTCGGCCA-3′
ferroportin	5′-TCAGTTTGCAACATGTCTGTACC-3′	5′-GCAACGTATTGCAGTCTCCAT-3′
SLC7A11	5′-TGACTGGAGTCCCTGCGTAT-3′	5′-TCTTCTTCTGGTACAACTTCCAGT-3′
GPX4	5’-CAGTGAGGCAAGACCGAAGT-3’	5’-CCGAACTGGTTACACGGGAA-3’
β-actin	5′-GGGCATGGGTCAGAAGGATT-3′	5′-TCGATGGGGTACTTCAGGGT-3′

## Data Availability

The raw data supporting the conclusions of this article will be made available by the authors on request.

## Supplementary Materials

The following supporting information can be downloaded at: https://www.mdpi.com/article/10.3390/biomedicines12102301/s1, Figure S1: The effect of ferroptosis inhibitors on Hcy-induced cell migration in HUVECs; Figure S2: Hcy suppressed the Akt/mTOR, MAPKs, and Nrf2/HO-1 signaling in HUVECs; Figure S3: Hcy suppressed the Akt/mTOR, MAPKs, and Nrf2/HO-1 signaling in HUVECs.
