# Supplementary figures and images for: Intracellular Iron Deficiency and Abnormal Metabolism, Not Ferroptosis, Contributes to Homocysteine-Induced Vascular Endothelial Cell Death
Source: Biomedicines. 2024 Oct 10;12(10):2301. doi: 10.3390/biomedicines12102301 (PMC11504269; doi:10.3390/biomedicines12102301)

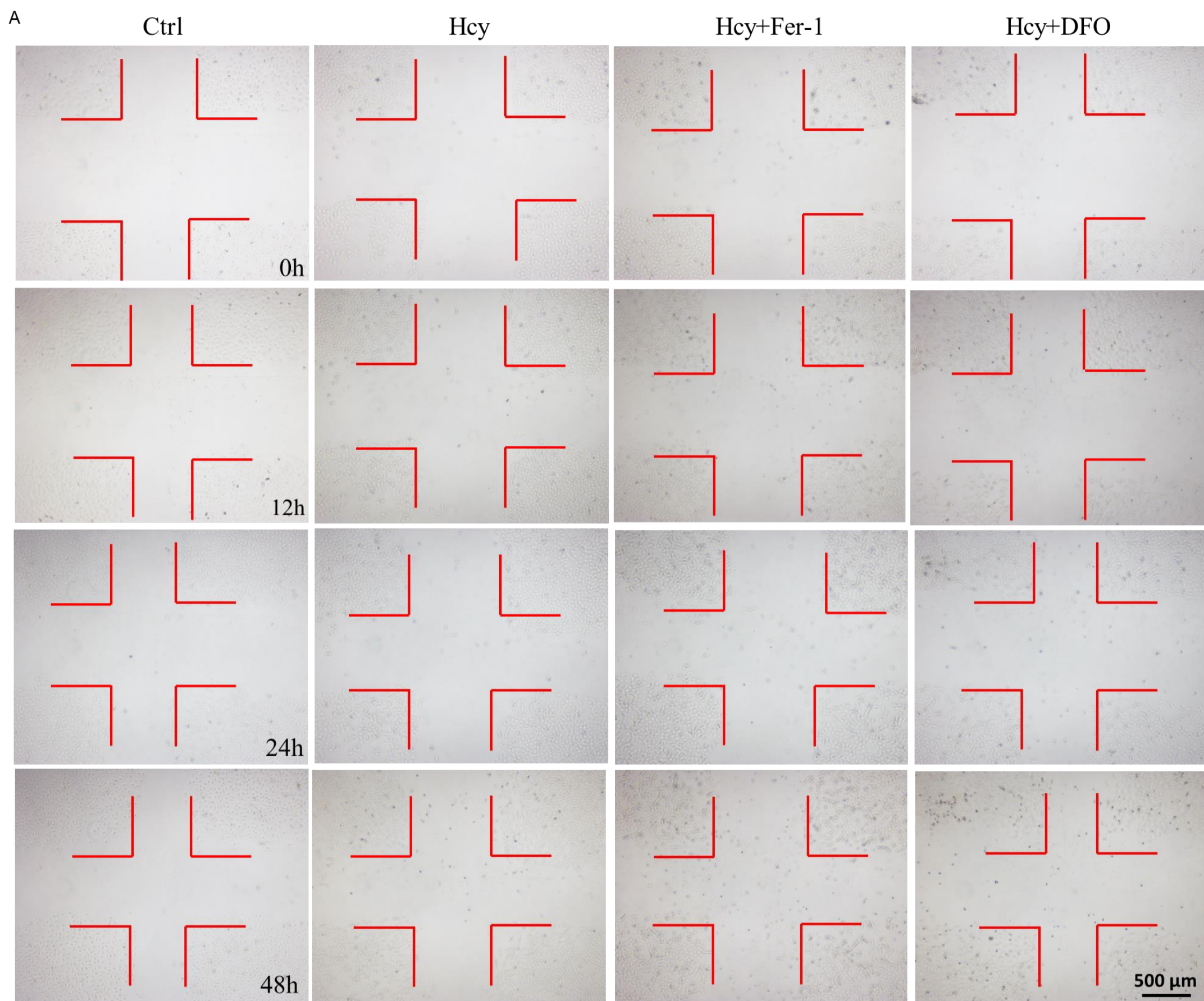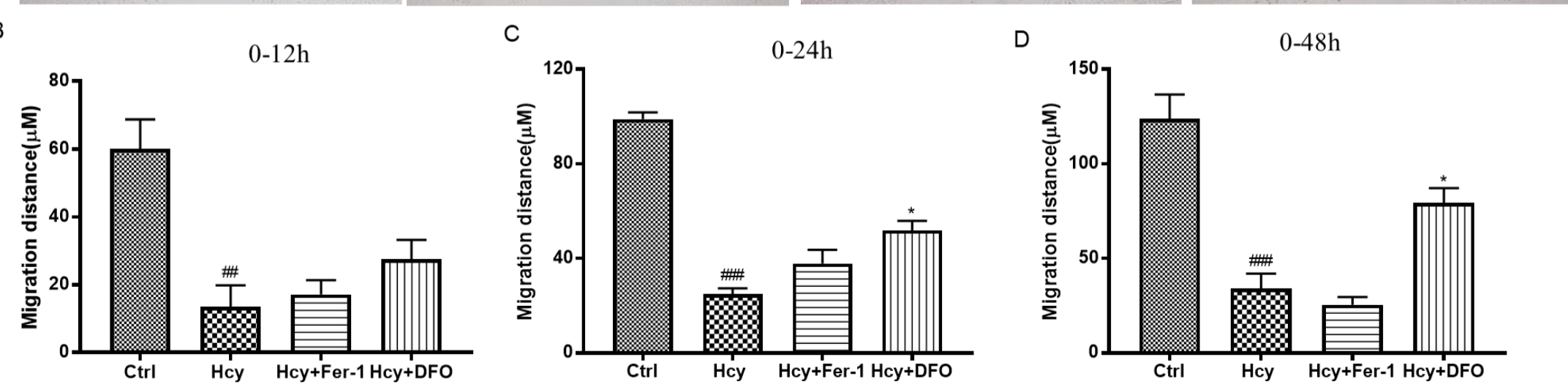

A

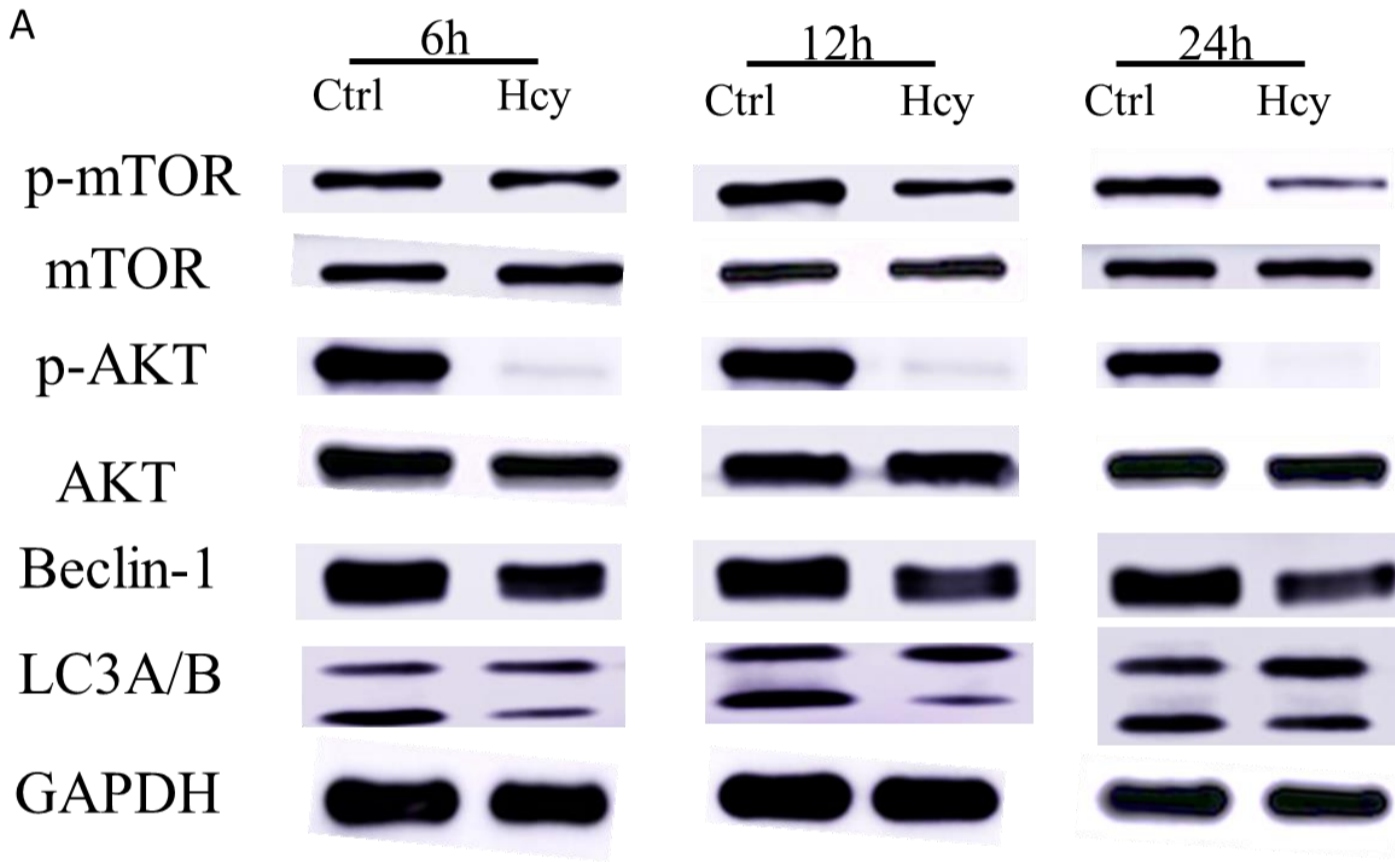

B

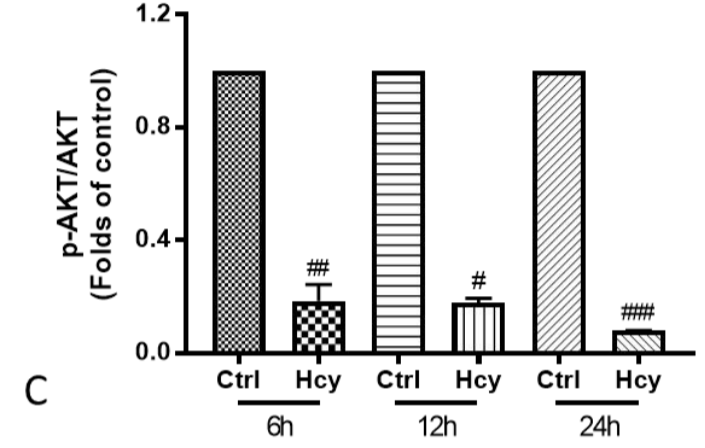

C

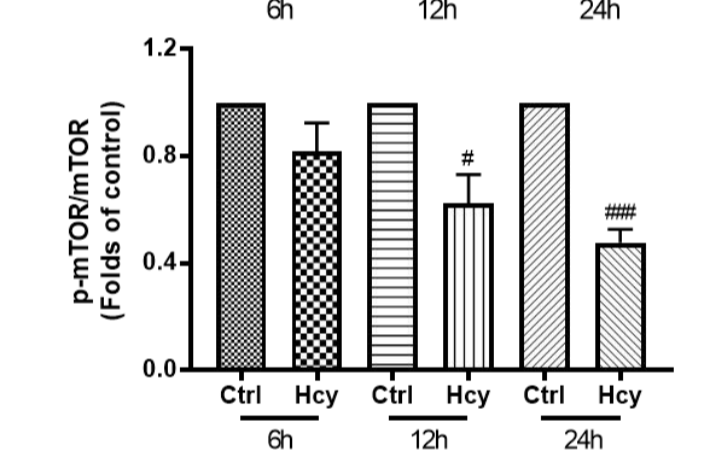

D

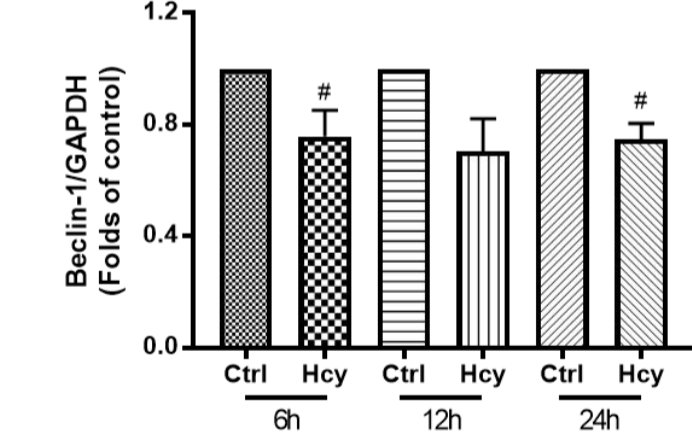

E

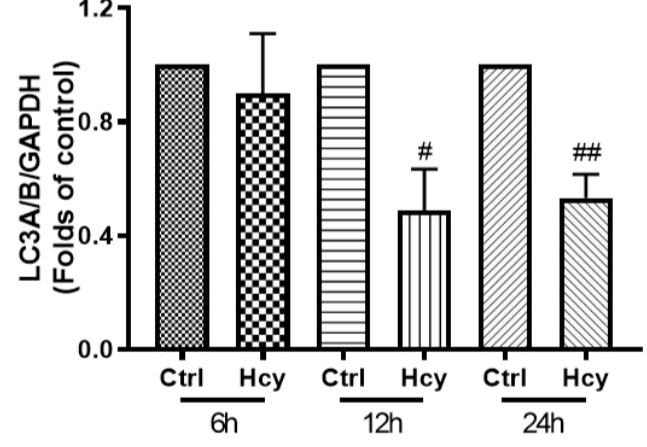

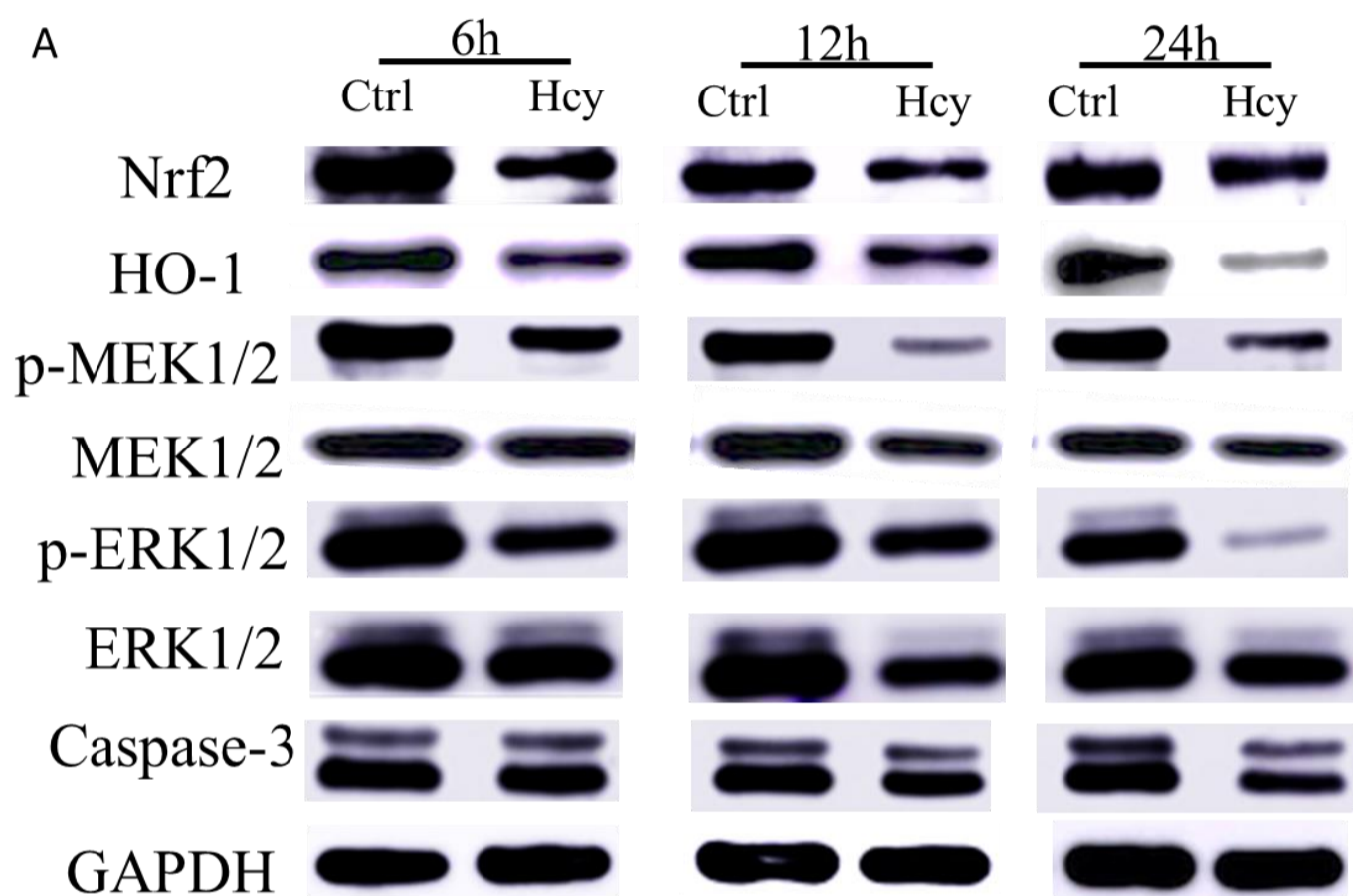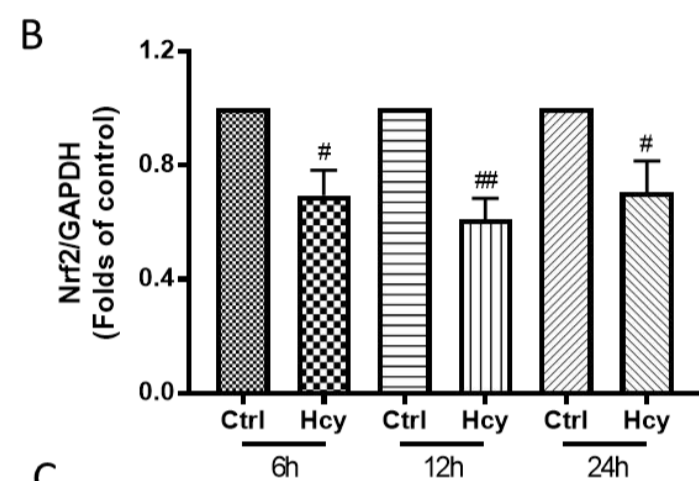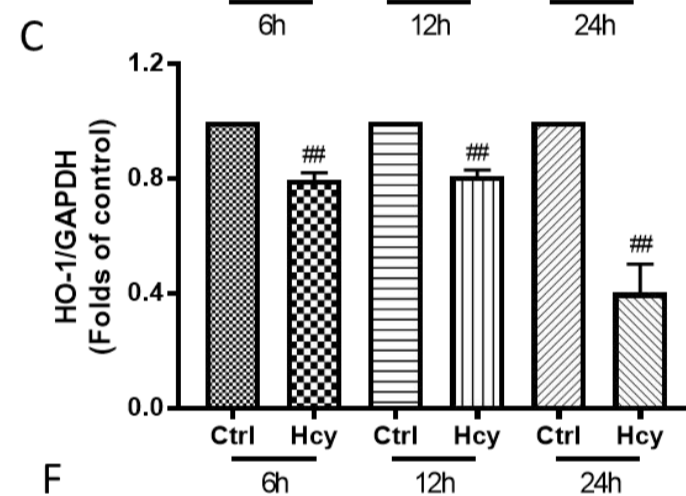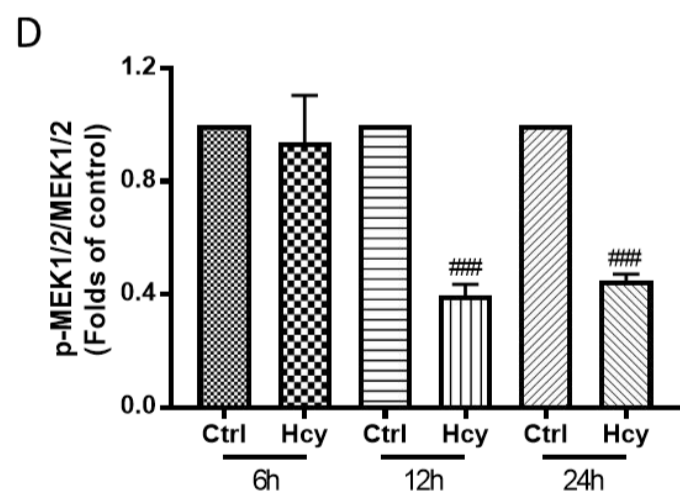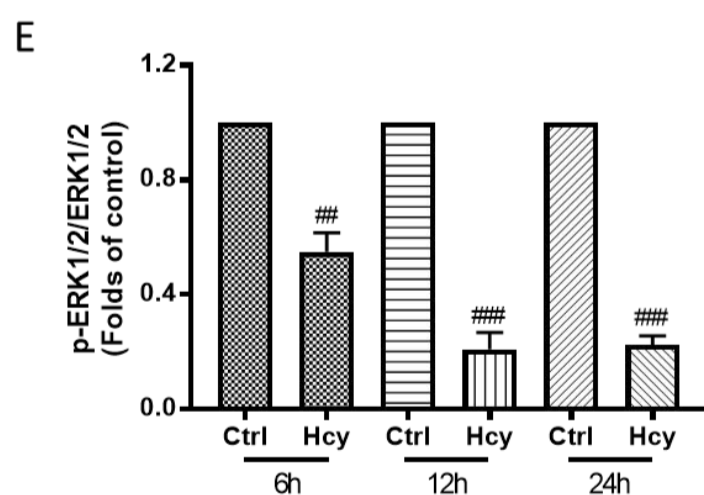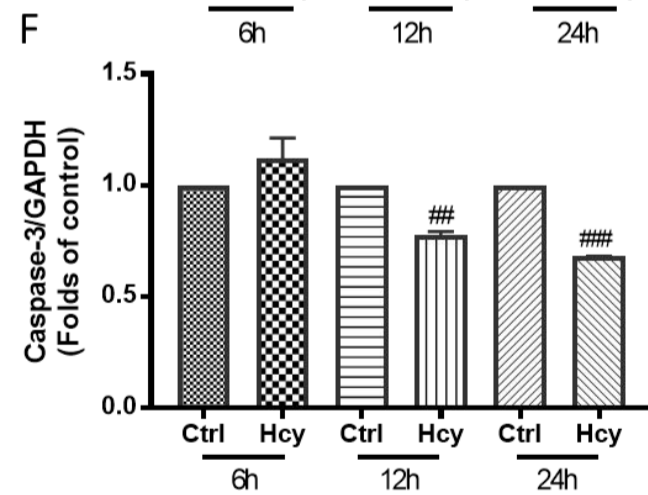

Supplement: Supplementary file 1 [file biomedicines-12-02301-s001.zip › biomedicines-3209666-supplementary.pdf]
